# Novel MRI technique for the quantification of biochemical deterioration in steroid-induced osteonecrosis of femoral head: a prospective diagnostic trial

**DOI:** 10.1093/jhps/hnab032

**Published:** 2021-06-17

**Authors:** Xiaorui Han, Guoju Hong, Yuan Guo, Hongzhen Wu, Ping Sun, Qiushi Wei, Zhenqiu Chen, Wei He, Zaiyi Liu, Changhong Liang

**Affiliations:** 1 Division of Biomedical Engineering, School of Medicine, South China University of Technology, Guangzhou, Guangdong 510006, China; 2 Department of Radiology, Guangdong Provincial People’s Hospital/Guangdong Academy of Medical Science, Guangzhou, Guangdong 510080, China; 3 Traumatology and Orthopedics Institute, Guangzhou University of Chinese Medicine, Guangzhou, Guangdong 510405, China; 4 Department of Orthopedic, The First Affiliated Hospital of Guangzhou University of Chinese Medicine, Guangzhou, Guangdong 510405, China; 5 Division of Orthopedic Surgery, University of Alberta, Edmonton, Alberta T6G 2R3, Canada; 6 Department of Radiology, Guangzhou First People’s Hospital/the Second Affiliated Hospital of South China University of Technology, Guangzhou, Guangdong, 510180, China; 7 Department of Endocrinology, The First Affiliated Hospital of Guangdong Pharmaceutical University, Guangzhou, Guangdong 510080, China

## Abstract

To explore the novel magnetic resonance imaging techniques, IVIM-DWI and IDEAL-IQ in detecting bone marrow fat and microcirculation in steroid-induced osteonecrosis of the femoral head (SIONFH). In this prospective study, 49 patients (80 hips) with SIONFH taking glucocorticoids and 24 healthy volunteers (48 hips) were recruited and assessed by T1WI, T2WI/*f*s, IDEAL-IQ and IVIM-DWI. The affected hips, contralateral asymptomatic hips and normal hips, as well as normal, penumbra and necrotic areas in the affected hips, were classified and evaluated. Imaging results were compared with histologic bone sections obtained from SIONFH patients undergoing surgery. The fat fraction (FF) and perfusion fraction (*f*) differences between groups were analyzed using analysis of variance, the LSD *t*-test, Pearson correlation analysis and ROC curve analysis. Our results demonstrate that IDEAL-IQ (FF) and IVIM-DWI (*f*) enable the classification of SIONFH at different ARCO stages. The FF was positively associated with the progression of the disease (*r* = 0.72), in contrast to *f* (*r* = −0.17). The FF and *f* were significantly different among the necrotic, penumbra and normal areas, and they were negatively correlated with each other (*r* = −0.37). The diagnostic sensitivity and specificity of IDEAL-IQ were 96.9% and 86.7%, and those of IVIM-DWI were 72.34% and 58.33%, respectively. The FF in contralateral asymptomatic hips was significantly higher than in normal hips, but no difference was found for *f*. IDEAL-IQ, and not IVIM-DWI, was identified to successfully detect bone marrow fat, which is beneficial to the diagnosis of the severity of SIONFH.

## INTRODUCTION

Steroid-induced osteonecrosis of the femoral head (SIONFH) is a chronic and devastating bone disease caused by prolonged and excessive glucocorticoid administration [1]. Glucocorticoid treatment leads to biochemical deterioration in the femoral head, including bone marrow fat deposition and poor perfusion of microcirculation, which is detected by histopathology [2]. Conventional morphological magnetic resonance imaging (MRI) is non-invasive and shows high accuracy in the detection of bone marrow osteonecrosis; however, it lacks the ability to detect biochemical deterioration of the femoral head in SIONFH [3]. It has been reported that 50–70% of cases of osteonecrosis of the femoral head (ONFH) have bilateral hip lesions [4–7], whereas the secondary hip is usually asymptomatic and shows no pathological signs in conventional MRI before the emergence of necrotic signs, possibly due to different susceptibilities of the two hips [8, 9]. Thus, even without osteonecrosis being detected, it is hypothesized that glucocorticoid induces biochemical deterioration in the asymptomatic hip. Because of the limitation of current MRI technology, such patients cannot be distinguished and undergo suitable treatments in advance [1, 10]. This poses great challenges with respect to the exploration of novel imaging techniques for the detection of biochemical substances in the femoral head.

In this prospective diagnostic trial, we used iterative decomposition of water and fat with echo asymmetry and least square estimation–iron quantification (IDEAL-IQ) and intravoxel incoherent motion–diffusion-weighted imaging (IVIM-DWI) as complementary techniques to assess the biochemical deterioration in SIONFH. IDEAL-IQ provides data of bone marrow fat content through detecting ‘in-phase’ and ‘out-phase’ images of water–fat signals [11]. Previous imaging studies revealed that IDEAL-IQ enables the quantitative analysis of the fat deposition in the bone marrow of different bone sites, such as the lumbar vertebral body, the ilium and the intertrochanteric region of the femur [11, 12]. IVIM-DWI accurately and non-invasively characterizes the microcirculation by detecting the diffusion and perfusion of water molecules in tissues [13]. Recent studies have demonstrated that IVIM-DWI performs excellently in the evaluation of bone marrow diffusion in multiple diseases, e.g. myeloma lesions and hepatocellular carcinoma [13–15]. Thus, both techniques may have the potential to be applied in osteonecrosis patients to examine the changes in biochemical composition of the bone marrow in the femoral head, particularly if no morphological features are observed.

Therefore, the purpose of this study is to explore the efficiency of IVIM-DWI and IDEAL-IQ in the evaluation of the bone marrow fat content and microcirculation in SIONFH, respectively. It is hypothesized that IVIM-DWI and IDEAL-IQ can help to detect the increased fat deposition and reduced perfusion in bone tissue and provide an early diagnosis of SIONFH.

## MATERIALS AND METHODS

### Participants

In total, 73 participants (41% male and 59% female; median age, 61 years; range, 45–79 years) were recruited from our institution between September 2017 and November 2018, i.e. (i) 49 participants (80 hips) who were diagnosed according to the *Chinese Guideline for the Diagnosis and Treatment of Osteonecrosis of the Femoral Head in Adults* [16] and evaluated using the ARCO classification (defined by conventional MRI prior to formal tests) (Supplementary Table SI) [17] and (ii) 24 healthy volunteers (48 hips). The characteristics of the participants are summarized in Supplementary Table SII. All SIONFH patients had received glucocorticoid treatment for more than 3 months (>2000 mg), were no excessive alcohol drinkers (alcohol addicts with a drinking history of at least 10 years) and had no hip trauma. Participants with ARCO Stage IV ONFH were excluded due to the mixed MRI signals in the femoral head, leading to possible misdiagnosis. Participants who had undergone surgical procedures for hip treatment prior to our research were also excluded. SIONFH individuals were identified within the institution from multiple referring specialties. This diagnostic prospective study was approved by the institutional review board in accordance with The Code of Ethics of the World Medical Association (Declaration of Helsinki) (Supplementary Table SIII). Written informed consent was obtained from each subject.

Three analysis strategies were used in our study. (i) The affected hips in the bilateral and unilateral cases were classified according to ARCO stage and compared with hips of the healthy volunteers. (ii) Affected hips were divided into three areas, i.e. the normal, penumbral and necrotic areas. (iii) The necrotic areas of the affected hips in the bilateral and unilateral cases were defined as the exposed group, the contralateral asymptomatic hips in the unilateral cases (in the absence of necrotic MRI signals) were defined as the risk group, and the hips of healthy volunteers were defined as the control group.

### MRI protocols

All individuals underwent MRI scanning of bilateral hip joints in the supine position using HDxt 3.0T magnetic resonance (Discovery MR750 3T, GE Healthcare, Milwaukee, WI, USA) equipped with a phase array coil (Philips Healthcare, The Netherlands). The lower extremities of the participants were fixed as hip extension and neutral rotation. In the sagittal position, the line of MRI acquisition was parallel to the femoral neck and passed through the center of the femoral head. Scans of both hips were obtained simultaneously. The scan line was set between the anterior-superior iliac spine and the pubic symphysis. The scanning sequences included sagittal and coronal T1-weighted imaging (T1WI), coronal fat-suppressed T2-weighted imaging (T2WI/*f*s), sagittal IDEAL-IQ and sagittal IVIM-DWI. The imaging parameters are presented in Table I.

**Table I.** MRI protocol for IDEAL-IQ and IVIM-DWI

MRI protocol		MRI sequence		
	T1WI	T2WI/fs	IDEAL-IQ	IVIM-DWI
Position	Sagittal/Coronal	Sagittal	Sagittal	Sagittal
TR (ms)	617	2900	4.8	5200
TE (ms)	16	1.3	1.5	68
FOV (mm × mm)	260 × 260	310 × 310	320 × 320	320 × 300
Matrix	512 × 288	288 × 224	384 × 512	320 × 300
Slice thickness (mm)	4	3	3	3
Slice gap (mm)	0.5	0.6	0.5	0.5
*b*-values (s/mm^2^)	/	/	/	10, 20, 30, 50, 80, 100, 200, 400, 800, 1000
Total scan time	11 min 15 s	4 min 6 s	2 min 34 s	3 min 12 s

FOV, field of view; TE, echo time; TR, repetition time.

**Fig. 1.**
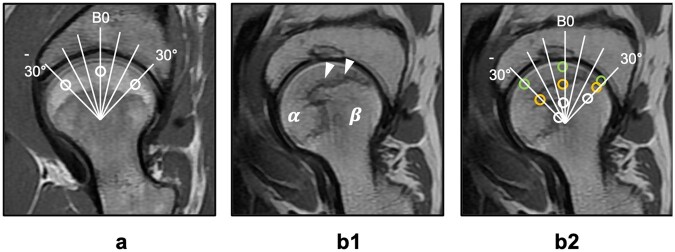
Division of the femoral head in T1WI images and ROI selection. The ROIs were used to divide the IDEAL-IQ and IVIM-DWI images of the femoral head into radial sections. (**a**) For the normal hips and contralateral asymptomatic hips, the center was confirmed using a concentric circle in the femoral head and a center line parallel to B0 that passed through the center of the femoral head (white circle: normal area selection). (**b1**) In SIONFH, the femoral head is divided into three parts, i.e. the normal area (β), penumbra area (white arrow, presenting as a low signal band) and necrotic area (α, presenting as a high signal). (**b2**) ROIs were allowed to be adjusted no more than 5° if the necrotic or penumbra area was not big enough (green circle: necrotic area selection; orange circle: penumbra area selection; white circle: normal area selection).

Imaging data of IDEAL-IQ and IVIM-DWI were post-processed using an AW4.5 workstation. In IDEAL-IQ, the pseudo-color image corresponding to the fat content was generated. Then we defined the region of interest (ROI) and calculated the fat fraction (FF) by the following formula: FF = (SI_IP_–SI_OP_)/(2SI_IP_). IVIM-DWI was performed using an MADC kit. The pseudo-color images were generated for matching the corresponding *b*-values (10–200 s/mm^2^), and then *f*, indicating perfusion of microcirculation, was obtained. The bone marrow FF and the microcirculation (*f*) were measured in each strategy. A center-weight-bearing line (parallel to the B0 line through the center of the femoral head) was set and ROIs were defined within the area ranging from −30° to +30° around the line. IDEAL-IQ and IVIM-DWI values were obtained and measured at the −30° line, the centerline and the +30° line in each area (Fig. 1a). ROIs were allowed to be adjusted no more than 5° if the necrotic or penumbra area was not big enough. This line drawing was also applied in the normal and risk groups (Fig. 1b and c). Each test was performed in triplicate and we recorded the averages as the final results. In order to ensure consistency between the tests, all of the steps were independently completed by two radiologists (X.R.H and Y.G., with 10 and 20 years of experience, respectively).

### Histologic evaluation and immunohistochemistry assay

Due to the indications of total hip arthroplasty for SIONFH, only patients at ARCO Stage III underwent surgery in our study (performed by Z.Q.C.). The necrotic femoral heads were collected for hematoxylin and eosin (H&E) staining and immunohistochemistry assay. Specifically, bone cubes were punched down from the normal, penumbral and necrotic areas of the isolated femoral head. Then the cubes were fixed, decalcified and embedded, and the created slices were stained using H&E. For immunohistochemistry, the slices were deparaffinized, rehydrated and incubated with anti-VEGF and a secondary antibody (Thermo Fisher Scientific, Waltham, MA, USA) (performed by G.J.H.).

### Statistical analysis

Statistical analyses were conducted using SPSS Statistics 22.0 (Chicago, IL, USA), except for the receiver operating characteristic (ROC) comparison, which was conducted using MedCalc (Ostend, Belgium). Both continuous and discrete variables are expressed as mean ± standard deviation (SD). The FF and *f* were compared between areas (normal, penumbra and necrotic areas) and groups (normal, exposed and risk groups) by analysis of variance (ANOVA). The LSD *t-*test was used for further multiple comparisons. The diagnostic sensitivity and specificity of the FF and *f* detected in the necrotic area were evaluated by ROC curve analysis.

## RESULTS

### Demographic characteristics

This study included 31 bilateral SIONFH cases (18 males and 13 females; median age, 43 years; range, 16–58 years) and 18 unilateral SIONFH cases (12 males and 6 females; median age, 42 years; range, 19–60 years). A total of 24 healthy volunteers without ONFH or any other hip joint disease were enrolled (14 males and 10 females; median age, 36; range, 19–47 years).

### Imaging of IDEAL-IQ and IVIM-DWI analysis

**Fig. 2.**
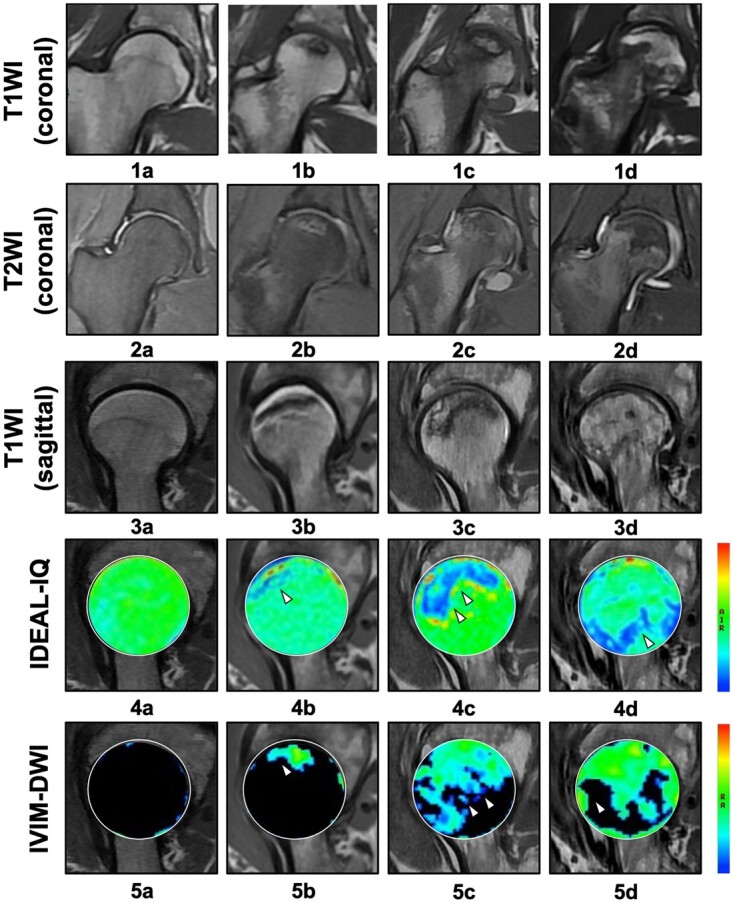
The progression of ARCO stage and signal changes in sagittal/coronal T1WI, sagittal T2WI/*f*s, sagittal IDEAL-IQ and sagittal IVIM-DWI sequences. (**a1–a5**) A 52-year-old female participant without SIONFH exhibited an even color gradation of the femoral head and clear boundaries at imaging. (**b1–b5**) A 53-year-old male ARCO Stage I patient showed a small blue band and a green color spot in the IDEAL-IQ and IVIM-DWI images, respectively. (**c1–c5**) A 61-year-old female ARCO Stage II patient exhibited mixed-color bands in both techniques. (**d1–d5**) A 41-year-old male ARCO Stage III patient showed a blue band indicating the penumbra area in IDEAL-IQ and diffuse and mixed colors (green–yellow–red) in IVIM-DWI.

**Fig. 3.**
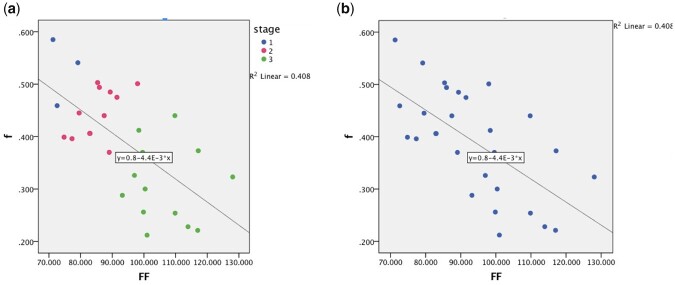
Relationships among IDEAL-IQ and IVIM-DWI and ARCO stage. (**a**) A positive correlation was observed between IDEAL-IQ and ARCO stage (*r* = 0.72, *P *=* *0.000). IVIM-DWI and ARCO stage were negatively correlated (*r* = −0.17, *P *=* *0.390). (**b**) IDEAL-IQ was negatively correlated with the IVIM-DWI (*r* = −0.37, *P *=* *0.048).

The pseudo-color images of IDEAL-IQ and IVIM-DWI were colonized through the spatial distributions of FF and *f*, both of which represent the dynamic changes of biochemical substances in the femoral head. When considering the IDEAL-IQ image, as shown in Fig. 2(a4), the femoral heads of healthy volunteers exhibited morphologic integrity, with a smooth articular surface and uniform green color gradation. In SIONFH ARCO Stages I and II, pseudo-color images containing unexpected yellow or red spots (necrotic area) and blue bands (penumbral area) were observed [Fig. 2(b4–c4)]. In SIONFH ARCO Stage III, these signs in the necrotic area progressively aggravated, presenting as fading or confused colors. Additionally, a large blue band was the main characteristic of the pseudo-color image of the penumbral area at this stage [Fig. 2(d4)]. The location of the necrotic area identified in T1WI and T2WI/*f*s images [Fig. 2(1–3)] was approximately consistent with the enhanced IDEAL-IQ signal, which was demonstrated by the positive correlation coefficient of 0.72 (*P *=* *0.000) between the FF and ARCO stages (Fig. 3a). In the necrotic area of the femoral head, the FF at ARCO Stages I–III gradually increased (increases of 28.63%, 47.66% and 83.37%, respectively, compared with normal hips; Table II); the statistical significance of these increases was confirmed by the LSD *t-*test (Table III). In IVIM-DWI, the observation of diffuse and mixed colors (green–yellow–red) in the femoral head indicated poor perfusion and ischemic injury. The area of patchy-like mixed colors was enlarged with the progression of the ARCO stage [Fig. 2(a5–d5)]. Although the level of microcirculation perfusion was negatively correlated with the fat deposition, as demonstrated by IDEAL-IQ (the correlation coefficient was −0.37, *P *=* *0.048; Fig. 3b), The locations of ischemic lesions [Fig. 2(a5–d5)] did not completely coincide with those of osteonecrosis shown in T1WI and T2WI/*f*s [Fig. 2(1–3)]. In the IVIM-DWI pseudo-color images, *f* decreased with the progression of the disease from Stages I to III [decreases of 17.54%, 21.44% and 45.6%, respectively (Table II)] and their correlation coefficient was −0.17 (*P *=* *0.390) (Fig. 3a). In the LSD *t-*test, the mean *f* of necrotic areas varied at each stage, but no significant difference was found between Stage I and Stage II (Table III).

**Table II.** IDEAL-IQ and IVIM-DWI in each stage of the femoral head

	ARCO stage				F value	P-value
	Normal (n = 48)	I (n = 12)	II (n = 28)	III (n = 40)		
FF (%)	57.80 ± 6.85	74.35 ± 4.21	85.35 ± 6.42	105.99 ± 0.05	59.02	0.000
F	0.57 ± 0.06	0.47 ± 0.03	0.37 ± 0.02	0.31 ± 0.07	37.07	0.000

**Table III.** LSD t-test for IDEAL-IQ and IVIM-DWI of multiple comparisons between ARCO stages

	ARCO stage		X ± s	P-value	95% CI
FF (%)	Normal	I	–16.55 ± 5.57	0.00	0.02, 0.18
		II	–27.55 ± 3.84	0.00	0.04, 0.15
		III	–48.20 ± 3.74	0.00	0.20, 0.31
	I	Normal	16.55 ± 5.57	0.00	–0.18, –0.02
		II	–11.00 ± 5.21	0.04	–0.08, 0.07
		III	–31.65 ± 5.13	0.00	0.08, 0.23
	II	Normal	27.55 ± 3.84	0.00	–0.15, –0.04
		I	11.00 ± 5.21	0.04	–0.07, 0.08
		III	–20.64 ± 3.18	0.00	0.11, 0.20
	III	Normal	48.20 ± 3.74	0.00	–0.31, –0.20
		I	31.65 ± 5.13	0.00	–0.23, –0.08
		II	20.64 ± 3.18	0.00	–0.20, –0.11
F	Normal	I	0.10 ± 0.04	0.02	–27.90, –5.21
		II	0.10 ± 0.03	0.00	–35.37, 19.73
		III	0.25 ± 0.03	0.00	–55.81, –40.59
	I	Normal	–0.10 ± 0.04	0.02	5.21, 27.9
		II	–0.00 ± 0.04	0.96	–21.61, –0.39
		III	0.16 ± 0.04	0.00	–42.10, –21.19
	II	Normal	–0.10 ± 0.03	0.00	19.73, 35.37
		I	0.00 ± 0.04	0.96	0.39, 21.61
		III	0.16 ± 0.02	0.00	40.59, –14.18
	III	Normal	–0.25 ± 0.03	0.00	40.59, 55.81
		I	–0.16 ± 0.04	0.00	21.19, 42.1
		II	–0.16 ± 0.02	0.00	14.18, 27.11

### Comparison of the IDEAL-IQ and IVIM-DWI values among different areas

To distinguish the appearances of different areas of the femoral head, the FF and *f* were further investigated at the normal, penumbral and necrotic areas in the femoral head of SIONFH patients. The average FF was enhanced by 102.42% at the necrotic area, whereas it was reduced to 38.56% at the penumbral area. The highest obtained value for *f* at the normal area was 0.4%, and lower values were obtained at the other areas (Tables IV and V).

**Table IV.** IDEAL-IQ and IVIM-DWI in each area of the femoral head

	Area of femoral head			F value	P-value
	NA (n = 80)	PA (n = 80)	OA (n = 80)		
FF (%)	75.24 ± 14.55	38.56 ± 13.04	102.42 ± 36.67	51.76	0.000
F	0.40 ± 0.08	0.34 ± 0.08	0.30 ± 0.07	12.36	0.000

Data are expressed as means ± SD.

FF, fraction of fat; NA, normal area; OA, osteonecrotic area; PA, penumbra area.

**Table V.** LSD t-test for IDEAL-IQ and IVIM-DWI of multiple comparisons between areas

ROI		X ± s	P-value	95% CI
FF (%)				
NA	OA	–27.18 ± 6.299	0.000	–39.71, –14.65
	PA	36.68 ± 6.299	0.000	24.15, 49.20
PA	OA	–63.86 ± 6.299	0.000	–76.38, –51.33
	NA	–36.68 ± 6.299	0.000	–49.20, –24.15
OA	PA	63.86 ± 6.299	0.000	51.33, 76.38
	NA	27.18 ± 6.299	0.000	14.65, 39.70
F				
NA	OA	0.10 ± 0.02	0.000	0.06, 0.14
	PA	0.06 ± 0.02	0.005	0.02, 0.10
PA	OA	0.04 ± 0.02	0.044	0.00, 0.08
	NA	–0.06 ± 0.02	0.005	–0.10, –0.02
OA	PA	–0.04 ± 0.02	0.044	–0.08, –0.00
	NA	–0.10 ± 0.02	0.000	–0.14, –0.06

Data are expressed as means ± SD.

FF, fraction of fat; NA, normal area; OA, osteonecrotic area; PA, penumbra area.

### Histological analysis of the femoral head

**Fig. 4.**
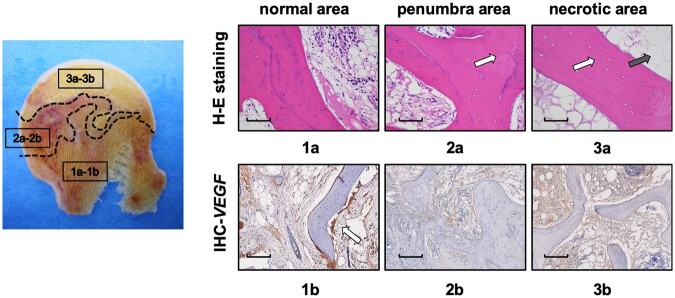
Histologic examination of the femoral head from patients undergoing total hip arthroplasty surgery. (**a**) Hard tissue slicing of the femoral head indicating different characteristics of the normal area (**a1–b1**, presenting as a yellow color), the penumbra area (**a2–b2**, presenting as a dark brown color with enhanced bone density) and the necrotic area (**a3–b3**, presenting as a cheese-like white color). Bone samples were punched down from these three areas. (**b**) H&E staining (a1–a3, the white arrow indicates the empty bone lacuna and the black arrow indicates adipocyte deposition; scale bar: 50 μm) and immunohistochemical staining of VEGF at the corresponding areas (**b1–b3**, the white arrow indicates the expression of VEGF on the periosteum; scale bar: 50 μm).

Histologic analysis of the femoral head from patients at ARCO Stage III was performed to analyse the categorical differences between areas of the femoral head. To identify the fat accumulation and ischemia of the femoral head by histomorphology, H&E staining and immunohistochemical staining against VEGF were performed in each part of the femoral head. H&E staining revealed the subtle evidence of cell death and karyopyknosis at the necrotic area, accompanied with an enhanced percentage of lipocyte accumulation in the bone marrow [Fig. 4(a1)]. By contrast, a high density of the trabecula and few lipocytes were observed in the penumbral area [Fig. 4(b1)]. VEGF was highly expressed in the normal bone environment, where it mainly contributes to endothelial cell differentiation and vascular formation. Our immunohistochemistry assay demonstrated that VEGF was highly expressed along the periosteum, the superficial layer of the trabecula at the normal area, indicative of vascularization of bone tissue [Fig. 4(a2)]. However, we found no evidence of VEGF expression at the penumbral and necrotic areas, which indicated ischemic injury resulting from coagulation in the vasculature or interruption of microcirculation [Fig. 4(b2–c2)]. Histologic assessment of femoral head specimens confirmed the quantitative analysis results of IDEAL-IQ and IVIM-DWI.

### Diagnostic value of IDEAL-IQ and IVIM-DWI for SIONFH by ROC curve analysis

**Fig. 5.**
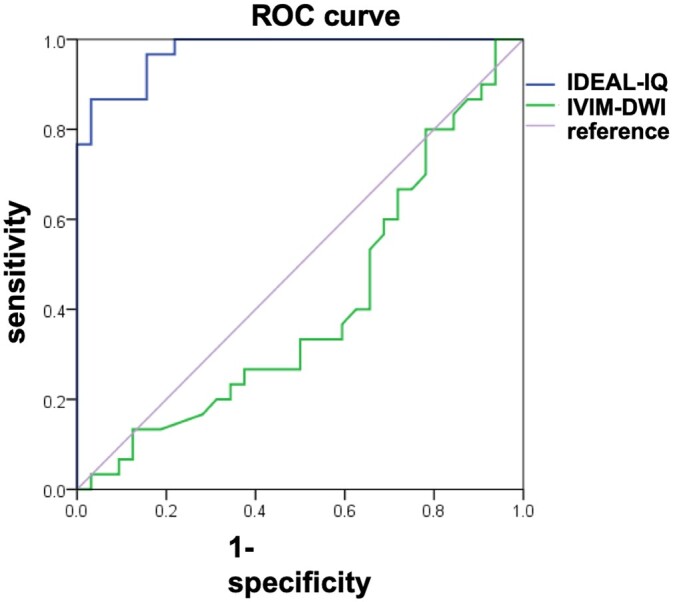
The AUC of IDEAL-IA (*Z *=* *31.52, *P *=* *0.031) is greater than that of IVIM-DWI (*Z *=* *1.25, *P *=* *0.021).

Taking the healthy controls and necrotic areas into account, IDEAL-IQ showed a considerably higher diagnostic value than IVIM-DWI. The area under the ROC curve (AUC) of the FF was 0.974, and its corresponding sensitivity and specificity were 96.9% and 86.7%, respectively, with a diagnostic threshold of 88.37 (*Z *=* *31.52, *P *=* *0.031) (Fig. 5). The AUC of *f* was 0.38, and its corresponding sensitivity and specificity were 59.4% and 66.7%, respectively, if a diagnostic threshold of 86.43 was selected (*Z *=* *1.25, *P *=* *0.021) (Fig. 5).

### Evaluation of sequences in asymptomatic hips of unilateral cases

**Fig. 6.**
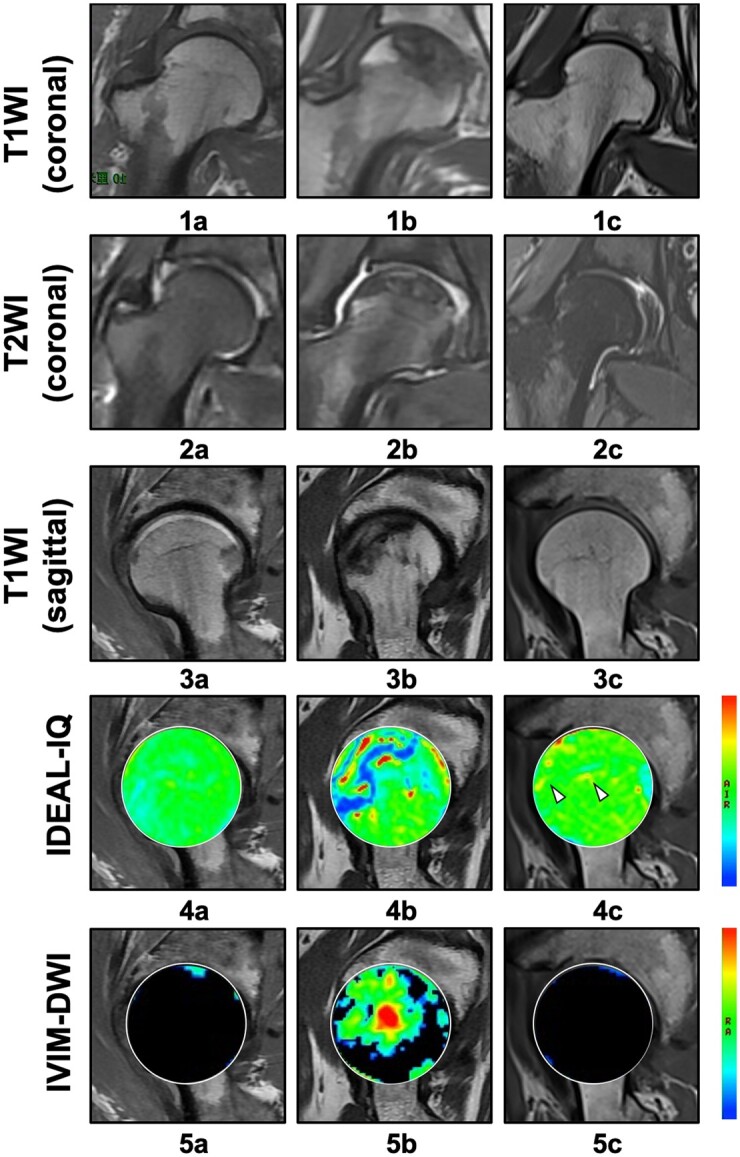
Signal changes of the femoral head of different groups in sagittal/coronal T1WI, sagittal T2WI/*f*s, sagittal IDEAL-IQ and sagittal IVIM-DWI sequences. (**a1–a5**) A 51-year-old female participant without SIONFH. (**b1–b5**) A 43-year-old male ARCO Stage III patient showed mixed signals with both techniques. (**c1–c5**) The contralateral asymptomatic hip of a unilateral 48-year-old male SIONFH patient exhibited blue color spots in IDEAL-IQ, but no particular feature was found by IVIM-DWI.

In order to analyze the diagnostic value of IDEAL-IQ and IVIM-DWI in asymptomatic hips, the hips in unilateral SIONFH patients (the risk group) were compared with the normal and exposed groups. The images of IDEAL-IQ showed slight changes with red and yellow spots in the femoral head in the risk group, in contrast to the normal group [Fig. 6 (a4–c4)]. Increases in FF of 14.11% and 26.36% in the risk and exposed groups supported the pseudo-color imaging results (Tables VI and VII). Nevertheless, in IVIM-DWI, no notable changes were detected in the pseudo-color images compared with the control group [Fig. 6 (a5–c5)], which was consistent with our statistical results with respect to *f* (ANOVA and LSD *t-*test) (Tables VI and VII).

**Table VI.** IDEAL-IQ and IVIM-DWI in each group of the femoral head

	Area of femoral head			F value	P-value
	NG (n = 48)	SG (n = 80)	RG (n = 18)		
FF (%)	80.88 ± 4.58	102.20 ± 36.05	92.29 ± 3.74	8.11	0.001
f	0.37 ± 0.07	0.31 ± 0.67	0.36 ± 0.06	8.93	0.000

Data are expressed as means ± SD.

EG, exposed group; FF, fraction of fat; NG, normal group; RG, risk group.

**Table VII.** LSD t-test for IDEAL-IQ and IVIM-DWI of multiple comparisons between groups

	Group		X ± s	P-value	95% CI
FF (%)	NG	EG	–9.91 ± 5.39	0.069	–20.62, 0.79
		RG	11.41 ± 5.30	0.034	0.87, 21.95
	EG	NG	9.91 ± 5.39	0.069	–0.79, 20.62
		RG	21.32 ± 5.30	0.000	10.79, 31.86
	RG	NG	–11.41 ± 5.30	0.034	–21.95, –0.87
		EG	–21.32 ± 5.30	0.000	–31.86, –10.79
f	NG	EG	0.05 ± 0.02	0.003	0.02, 0.08
		RG	–0.02 ± 0.02	0.337	–0.05, 0.02
	EG	NG	–0.05 ± 0.02	0.003	–0.08, –0.02
		RG	–0.07 ± 0.02	0.000	–0.10, –0.03
	RG	NG	0.02 ± 0.02	0.337	–0.02, 0.05
		EG	0.07 ± 0.02	0.000	0.03, 0.10

Data are expressed as means ± SD.

EG, exposed group; FF, fraction of fat; NG, normal group; RG, risk group.

## DISCUSSION

This prospective diagnostic study shows high patient-based sensitivity of quantitative IDEAL-IQ, and not IVIM-DWI, in the detection of the biochemical deterioration of the femoral head in SIONFH. Our findings demonstrate that the bone marrow fat content and microcirculation vary in different areas of the necrotic femoral head, which is consistent with the uneven lipid distribution and the intravascular obstruction observed in histological evaluation. Firstly, the FF is significantly higher in the necrotic area than in other areas, presumably due to the fat mobilization stimulated by the excessive glucocorticoid administration [2]. The active lipid metabolism causes an increase in the number of adipocytes in the femoral head, resulting in fat embolization in the microcirculation and fat deposition in osteocytes [18, 19]. Interestingly, the FF was the lowest in the penumbra area. It is assumed that lipid accumulation and high stress occur in the necrotic area, deeply compressing the penumbra area and increasing the trabecular density, leaving less space for fat deposition [20, 21]. A lack of angiogenesis in the femoral head is regarded as the main cause of ischemic injury in SIONFH. Bone vascularization can be re-established in SIONFH if treated with mesenchymal stem cells, activating tissue angiogenesis [22]. Anticoagulants like enoxaparin and warfarin have been found to improve blood supply and prevent the development of SIONFH [23]. In this study, *f* was lower in the necrotic and penumbra areas, suggesting poor perfusion of microcirculation due to excessive glucocorticoid administration [18].

Furthermore, our study revealed a high concordance between the biochemical deterioration of the femoral head and the progression of the ARCO stage. Specifically, with the aggravation of SIONFH, bone marrow fat accumulated, and microcirculation perfusion was partially blocked in the femoral head. The FF and *f* in the necrotic area were negatively correlated. Both observations support the idea that interruption or coagulation of the microvasculature in the femoral head results from fat deposition [18]. However, it is surprising that the diagnostic value of IDEAL-IQ in detecting SIONFH is much higher than that of IVIM-DWI, according to the ROC analysis results. Based on the low diagnostic value of IVIM-DWI, IVIM-DWI cannot provide evidence to support the downregulated signals in the corresponding images. Therefore, we speculate that IVIM-DWI is not sensitive enough to examine the microcirculation in the femoral head, although it is widely believed that poor perfusion often results from fat deposition.

Another objective of this research was to investigate the biochemical characteristics in the contralateral asymptomatic hips of unilateral cases by these two techniques. We found that the FF of the risk group was higher than that of the normal group and much lower than that of the exposed group. The ability of IDEAL-IQ to detect changes in biochemical substances in the asymptomatic hip probably anticipates active signals or morphological changes observed in T1WI and T2WI/*f*s, even though a long-term follow-up study is required to confirm this hypothesis. Furthermore, it is assumed that the lipid disorder, the primary pathomechanism induced by glucocorticoid treatment, occurs much earlier than microvascular coagulation [18, 19]. This is supported by the insignificant difference with respect to *f* between groups.

Currently, quantitative analysis of bone marrow fat and microcirculation in the femoral head is still not fully developed. Some limitations should be taken into consideration in our study. First, IVIM-DWI is rarely used in the quantitative evaluation of musculoskeletal microcirculation, whereas IDEAL-IQ has been mainly utilized in vertebrae in previous studies [11, 12]. Therefore, the applications of both techniques in SIONFH are immature and require further exploration. Second, a low number of bone samples from patients at ARCO Stage III were collected for histological examination, because often ARCO Stages I and II patients prefer hip preservation treatment [16, 24]. Third, ROIs in the different areas of the femoral head were manually delineated. The measurement of IDEAL-IQ and IVIM-DWI values is susceptible to be distorted if the pseudo-color images of the femoral head are difficult to interpret; it is almost impossible to avoid the random errors that occur when sketching an ROI. Fourth, IVIM-DWI is unable to detect microvascular coagulation or perfusion based on our results. Hence, the exploration of substitute radiological technologies, such as T2*-weighted dynamic susceptibility contrast or T1-weighted dynamic contrast enhancement, is expected in the near future, even though most of these techniques require intravenous injection of imaging agents and have never been used in SIONFH [25, 26].

Taking these facts into account, the high sensitivity and specificity of the non-invasive IDEAL-IQ technique is promising for the detection of the deterioration of bone marrow and the measurement of fat content in the femoral head. The detection of microcirculation perfusion, by contrast, needs further exploration.

## SUPPLEMENTARY DATA

Supplementary data are available at *Journal of Hip Preservation Surgery* online.

## FUNDING

National Natural Science Foundation of China (Contract Grant Number: 81925023, 81771912, 82071892); the Key R&D Program of Guangdong Province (Contract Grant Number: 2018B030339001); the National Key R&D Program of China (Contract Grant Number: 2017YFC1309100); the National Science Fund for Distinguished Young Scholars (Contract Grant Number: 81925023) and Science and Technology Planning Project of Guangdong Province (Contract Grant Number: 2017B020227012). 

## ETHICAL APPROVAL

This diagnostic prospective study was approved by the institutional review board in accordance with The Code of Ethics of the World Medical Association (No. ZYYECK[2017]126). Chinese Clinical Trail Registry, No. ChiCTR-RPC-15006290.

## Authors’ contributions

C.L. and G.H. conceived and supervised the study; G.H. and X.H. designed experiments; X.H., Y.G. and H.W. performed experiments; P.S. and Q.W. provided new tools and reagents; W.H. performed surgery on enrolled subjects; Q.W., X.C. and Z.L. analyzed data; G.H. and X.H. wrote the manuscript and G.H. and C.L. made manuscript revisions.

## CONFLICT OF INTEREST STATEMENT

None declared. 

## Supplementary Material

hnab032_Supplementary_Data

## References

[1] Liu LH, Zhang QY, Sun W et al Corticosteroid-induced osteonecrosis of the femoral head: detection, diagnosis, and treatment in earlier stages. Chin Med J 2017; 130: 2601–7. 29067959 10.4103/0366-6999.217094 PMC5678261

[2] Wang A, Ren M, Wang J. The pathogenesis of steroid-induced osteonecrosis of the femoral head: a systematic review of the literature. Gene 2018; 671: 103–9. 29859289 10.1016/j.gene.2018.05.091

[3] Pierce TP, Jauregui JJ, Cherian JJ et al Imaging evaluation of patients with osteonecrosis of the femoral head. Curr Rev Musculoskelet Med 2015; 8: 221–7. 26045084 10.1007/s12178-015-9279-6 PMC4596197

[4] Fukushima W, Fujioka M, Kubo T et al Nationwide epidemiologic survey of idiopathic osteonecrosis of the femoral head. Clin Orthop Relat Res 2010; 468: 2715–24. 20224959 10.1007/s11999-010-1292-x PMC2939331

[5] Tsai SW, Wu PK, Chen CF et al Etiologies and outcome of osteonecrosis of the femoral head: etiology and outcome study in a Taiwan population. J Chin Med Assoc 2016; 79: 39–45. 26387635 10.1016/j.jcma.2015.07.010

[6] Koo KH, Kim R, Kim YS et al Risk period for developing osteonecrosis of the femoral head in patients on steroid treatment. Clin Rheumatol 2002; 21: 299–303. 12189457 10.1007/s100670200078

[7] Zhao FC, Hu HX, Zheng X et al Clinical analysis of 23 cases of steroid-associated osteonecrosis of the femoral head with normal initial magnetic resonance imaging presentation. Medicine 2017; 96: e8834. 29245246 10.1097/MD.0000000000008834 PMC5728861

[8] Sonoda K, Yamamoto T, Motomura G et al Contralateral osteonecrosis of the femoral head newly developed after increasing the dose of corticosteroids. J Orthop Sci 2015; 20: 772–5. 24510362 10.1007/s00776-014-0541-3

[9] Sonoda K, Yamamoto T, Motomura G et al Bilateral corticosteroid-induced osteonecrosis of the femoral head detected at a 6-week interval. Springerplus 2015; 4: 662. 26558165 10.1186/s40064-015-1458-9 PMC4630241

[10] Baig SA, Baig MN. Osteonecrosis of the femoral head: etiology, investigations, and management. Cureus 2018; 10: e3171. 30357068 10.7759/cureus.3171 PMC6197539

[11] Aoki T, Yamaguchi S, Kinoshita S et al Quantification of bone marrow fat content using iterative decomposition of water and fat with echo asymmetry and least-squares estimation (IDEAL): reproducibility, site variation and correlation with age and menopause. Br J Radiol 2016; 89: 20150538. 27356277 10.1259/bjr.20150538 PMC5124909

[12] Hu L, Zha YF, Wang L et al Quantitative evaluation of vertebral microvascular permeability and fat fraction in alloxan-induced diabetic rabbits. Radiology 2018; 287: 128–36. 29156149 10.1148/radiol.2017170760

[13] Yoon MA, Hong SJ, Lee CH et al Intravoxel incoherent motion (IVIM) analysis of vertebral bone marrow changes after radiation exposure from diagnostic imaging and interventional procedures. Acta Radiol 2017; 58: 1260–8. 28103708 10.1177/0284185116688380

[14] Baik JS, Jung JY, Jee WH et al Differentiation of focal indeterminate marrow abnormalities with multiparametric MRI. J Magn Reson Imaging 2017; 46: 49–60. 27859835 10.1002/jmri.25536

[15] Bourillon C, Rahmouni A, Lin C et al Intravoxel incoherent motion diffusion-weighted imaging of multiple myeloma lesions: correlation with whole-body dynamic contrast agent-enhanced MR imaging. Radiology 2015; 277: 773–83. 26131910 10.1148/radiol.2015141728

[16] Microsurgery Department of the Orthopedics Branch of the Chinese Medical Doctor Association, Group from the Osteonecrosis and Bone Defect Branch of the Chinese Association of Reparative and Reconstructive Surgery, and Microsurgery and Reconstructive Surgery Group of the Orthopedics Branch of the Chinese Medical Association. Chinese guideline for the diagnosis and treatment of osteonecrosis of the femoral head in adults. Orthoped Surg 2017; 9: 3–12. 10.1111/os.12302 PMC6584093 28371498

[17] Sultan AA, Mohamed N, Samuel LT et al Classification systems of hip osteonecrosis: an updated review. Int Orthop 2019; 43: 1089–95. 29916002 10.1007/s00264-018-4018-4

[18] Zhang Q, L VJ, Jin L. Role of coagulopathy in glucocorticoid-induced osteonecrosis of the femoral head. J Int Med Res 2018; 46: 2141–8. 28459353 10.1177/0300060517700299 PMC6023042

[19] Shah KN, Racine J, Jones LC et al Pathophysiology and risk factors for osteonecrosis. Curr Rev Musculoskelet Med 2015; 8: 201–9. 26142896 10.1007/s12178-015-9277-8 PMC4596210

[20] Ma JX, He WW, Zhao J et al Bone microarchitecture and biomechanics of the necrotic femoral head. Sci Rep 2017; 7: 13345. 29042586 10.1038/s41598-017-13643-2 PMC5645321

[21] Di Benedetto P, Niccoli G, Beltrame A et al Histopathological aspects and staging systems in non-traumatic femoral head osteonecrosis: an overview of the literature. Acta Biomed 2016; 87(Suppl. 1): 15–24. 27104316

[22] Liu X, Li Q, Niu X et al Exosomes secreted from human-induced pluripotent stem cell-derived mesenchymal stem cells prevent osteonecrosis of the femoral head by promoting angiogenesis. Int J Biol Sci 2017; 13: 232–44. 28255275 10.7150/ijbs.16951 PMC5332877

[23] Guo P, Gao F, Wang Y et al The use of anticoagulants for prevention and treatment of osteonecrosis of the femoral head: a systematic review. Medicine 2017; 96: e6646. 28422866 10.1097/MD.0000000000006646 PMC5406082

[24] Yu X, Zhang D, Chen X et al Effectiveness of various hip preservation treatments for non-traumatic osteonecrosis of the femoral head: a network meta-analysis of randomized controlled trials. J Orthop Sci 2018; 23: 356–64. 29291916 10.1016/j.jos.2017.12.004

[25] Khalifa F, Soliman A, El-Baz A et al Models and methods for analyzing DCE-MRI: a review. Med Phys 2014; 41: 124301. 25471985 10.1118/1.4898202

[26] Sourbron SP, Buckley DL. Classic models for dynamic contrast-enhanced MRI. NMR Biomed 2013; 26: 1004–27. 23674304 10.1002/nbm.2940

