# Supplementary material for: Novel MRI technique for the quantification of biochemical deterioration in steroid-induced osteonecrosis of femoral head: a prospective diagnostic trial
Source: J Hip Preserv Surg. 2021 Jun 17;8(1):40–50. doi: 10.1093/jhps/hnab032 (PMC8460153; doi:10.1093/jhps/hnab032)
Supplement: hnab032_Supplementary_Data [file hnab032_supplementary_data.zip › Sup 1-2.docx]

**Sup 1.** The ARCO International Classification of Osteonecrosis of the Femoral Head

| STAGE | 0 | I | II | III | IV |
| --- | --- | --- | --- | --- | --- |
| FINDINGS | All present techniques normal or non-diagnostic | X-ray and CT are normal at least ONE of the below mentioned is positive | NO CRESENT SIGN  X-RAY ABNORMAL,  Sclerosis, osteolysis, focal porosis | CRESCENT SIGN  on the X-ray and/or flattening of articular surface of femoral head | OSTEOARTHRITIS  Joint space narrowing, acetabular changes, joint destruction |
| TECHNIQUES | X-ray, CT, Scintigraph, MRI | Scintigraph, MRI, *QUANTITATE on MRI | X-ray, CT, Scintigraph MRI  *QUANTITATE MRI & X-ray | X-ray, CT only  *QUANTITATE on X-ray | X-ray ONLY |
| SUBCLASSIFICATION | NO | lesions subdivided into medial, central, or lateral depending on location of involvement of femoral head | | | NO |
| QUANTITATION | NO | II-A < 15% involvement of femoral head  II-B 15–30% involvement of femoral head  II-C >30% involvement of femoral head | | III-A < 15% involvement of femoral head or <2mm depression of femoral head  III-B 15–30% involvement of femoral head or 2–4 mm depression of femoral head  III-C > 30% involvement of femoral head or > 4 mm depression of femoral head | NO |

Note. ARCO, Association Research Circulation Osseous

Cited from Gardeniers J.W.M., Gosling-Gardeniers A.C., Rijnen W.H.C. (2014) The ARCO Staging System: Generation and Evolution Since 1991. In: Koo KH., Mont M., Jones L. (eds) Osteonecrosis. Springer, Berlin, Heidelberg

**Sup 2,** Characteristics of SIONFH and non-SIONFH evolved in the research

| Patients characteristics | SIONFH in bilateral hips | SIONFH in unilateral hip | Healthy volunteers |
| --- | --- | --- | --- |
| Cases (n) | 31 | 18 | 24 |
| Number of hips (n) | 62 | 18 | 48 |
| Median Age (range) | 43 (16-58) | 42 (19-60) | 36 (19-47) |
| Males: females (no.) | 18:13 | 12:16 | 14:10 |
| Weight (kg) | 57.8±5.2 | 61.2±7.3 | 56.2±3.2 |
| I stage (n) | 9 | 3 | NA |
| II stage (n) | 23 | 5 | NA |
| III stage (n) | 30 | 10 | NA |
