# Supplementary figures and images for: Novel MRI technique for the quantification of biochemical deterioration in steroid-induced osteonecrosis of femoral head: a prospective diagnostic trial
Source: J Hip Preserv Surg. 2021 Jun 17;8(1):40–50. doi: 10.1093/jhps/hnab032 (PMC8460153; doi:10.1093/jhps/hnab032)

**Sup 3,** Ethical approval letter


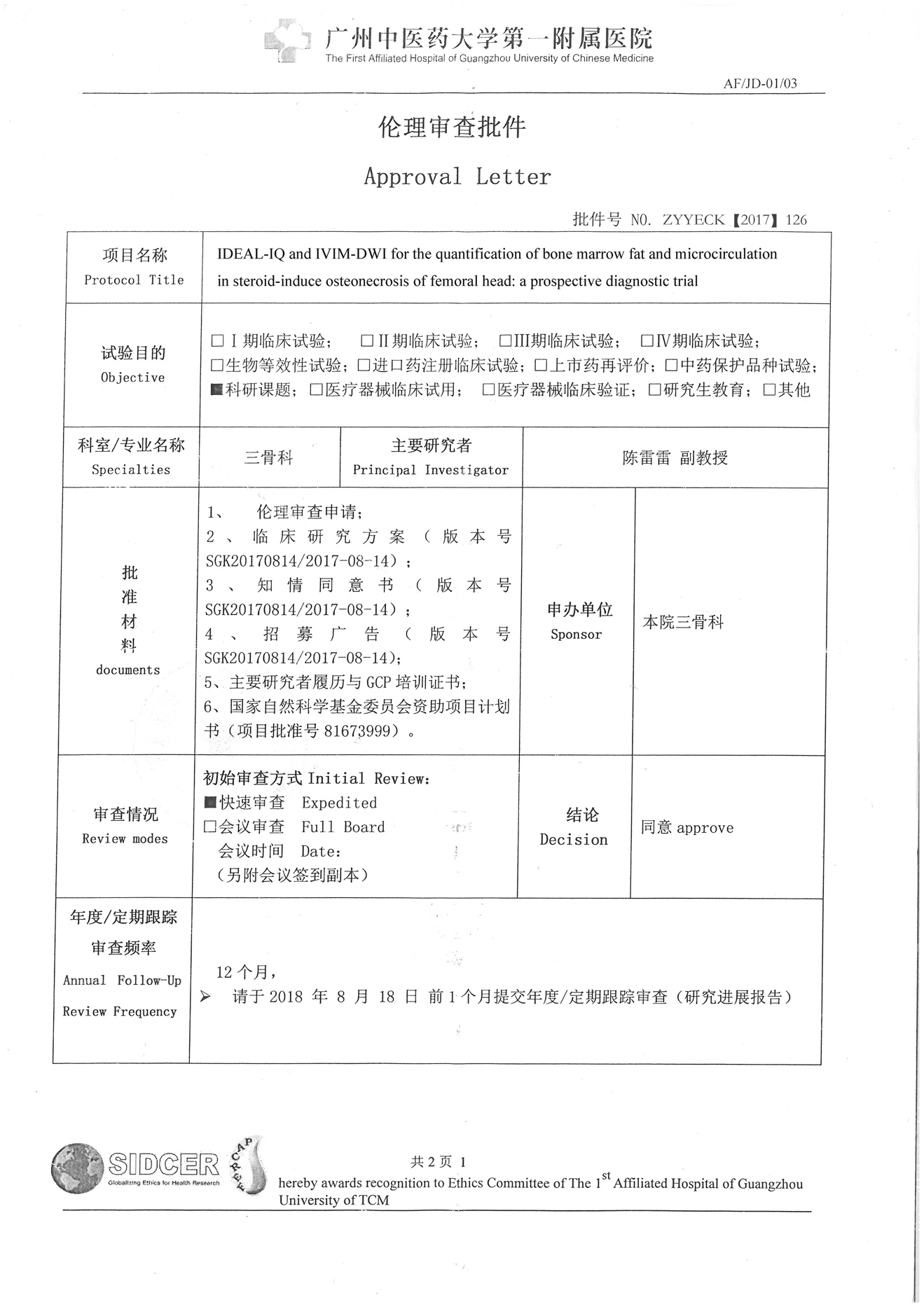


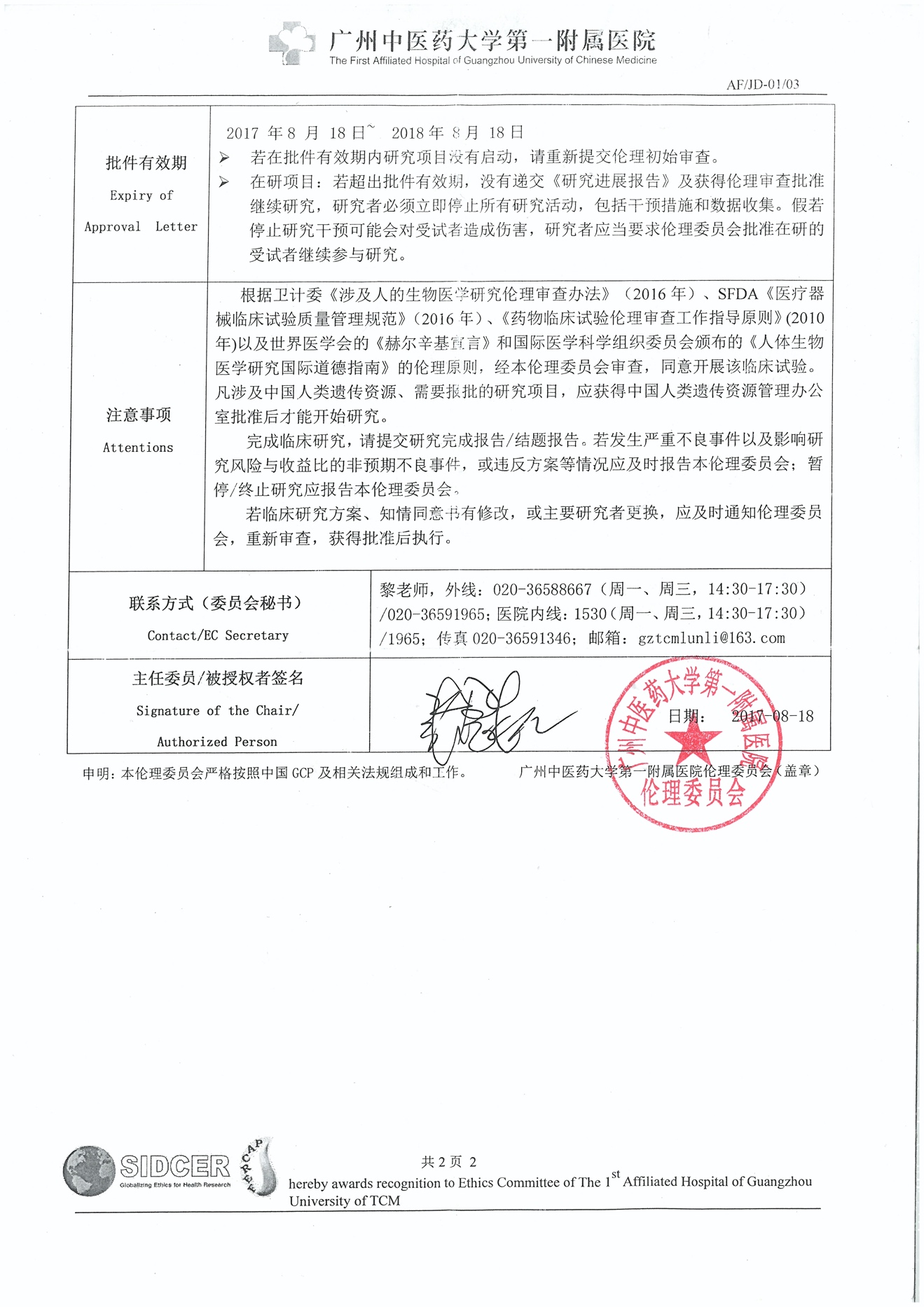

Supplement: hnab032_Supplementary_Data [file hnab032_supplementary_data.zip › Sup 3.docx]
